# Immigration Rates in Fragmented Landscapes – Empirical Evidence for the Importance of Habitat Amount for Species Persistence

**DOI:** 10.1371/journal.pone.0027963

**Published:** 2011-11-18

**Authors:** Thomas Püttker, Adriana A. Bueno, Camila dos Santos de Barros, Simone Sommer, Renata Pardini

**Affiliations:** 1 Departamento de Zoologia, Instituto de Biociências, Universidade de São Paulo, São Paulo, Brazil; 2 Departamento de Ecologia, Instituto de Biociências, Universidade de São Paulo, São Paulo, Brazil; 3 Department of Evolutionary Genetics, Leibniz Institute for Zoo and Wildlife Research, Berlin, Germany; Texas A&M University, United States of America

## Abstract

**Background:**

The total amount of native vegetation is an important property of fragmented landscapes and is known to exert a strong influence on population and metapopulation dynamics. As the relationship between habitat loss and local patch and gap characteristics is strongly non-linear, theoretical models predict that immigration rates should decrease dramatically at low levels of remaining native vegetation cover, leading to patch-area effects and the existence of species extinction thresholds across fragmented landscapes with different proportions of remaining native vegetation. Although empirical patterns of species distribution and richness give support to these models, direct measurements of immigration rates across fragmented landscapes are still lacking.

**Methodology/Principal Findings:**

Using the Brazilian Atlantic forest marsupial Gray Slender Mouse Opossum (*Marmosops incanus*) as a model species and estimating demographic parameters of populations in patches situated in three landscapes differing in the total amount of remaining forest, we tested the hypotheses that patch-area effects on population density are apparent only at intermediate levels of forest cover, and that immigration rates into forest patches are defined primarily by landscape context surrounding patches. As expected, we observed a positive patch-area effect on *M. incanus* density only within the landscape with intermediate forest cover. Density was independent of patch size in the most forested landscape and the species was absent from the most deforested landscape. Specifically, the mean estimated numbers of immigrants into small patches were lower in the landscape with intermediate forest cover compared to the most forested landscape.

**Conclusions/Significance:**

Our results reveal the crucial importance of the total amount of remaining native vegetation for species persistence in fragmented landscapes, and specifically as to the role of variable immigration rates in providing the underlying mechanism that drives both patch-area effects and species extinction thresholds.

## Introduction

The effects of patch size and isolation on populations have been intensively investigated, providing empirical evidence of their influence on demographic parameters, such as population size [1], [2], survival rates [3]–[5], and extinction probabilities [2], [6]. However, studies are usually conducted in single landscapes, despite the growing recognition that population dynamics in patches cannot be considered in isolation of the wider landscape context [7], [8].

Among whole-landscape properties, the total amount of remaining native vegetation (i.e. the amount of original, naturally occurring vegetation type) is expected to be of particular importance to populations in patches due to its non-linear relationships to patch- as well as gap-characteristics [9]–[12]. Simulation models suggest that the number of patches as well as the total amount of edge habitat is highest in landscapes with intermediate native vegetation cover, while the size of the largest patch decreases dramatically at around 60% habitat cover [9], [11]. By contrast, gap characteristics such as the average distances among neighboring patches increase exponentially below ∼10–20% habitat cover [9], [11] and therefore should have a strong influence on patterns of dispersal [13]–[15], increasing population isolation and decreasing the probability of rescue effects [16]. Small population sizes coupled with limited dispersal can increase vulnerability to local extinctions [17] and ultimately increase extinction probabilities at the landscape scale (i.e. species extinction thresholds; [13], [18]–[20]).

In linking the interaction between landscape context with patch-area effects, Andrén [9] and more recently Pardini *et al*. [20] proposed theoretical models that include a threshold in the total amount of remaining native vegetation, below which a positive patch-area effect on population density becomes apparent due to the increased isolation of patches. In this model the isolation of patches and consequent changes in immigration rates are defined by the total amount of native vegetation at the landscape scale [20]. According to the model, a moderate reduction in habitat cover should only affect populations in small patches, which due to their small size are more dependent on immigration to ensure population persistence, leading to a patch-area effect [9], [20]. By contrast a high level of vegetation clearance drives an exponential increase in distances among patches [10], leading to a drastic reduction in immigration rates and an increase in extinction risk within even the largest remaining patches [20].

Following a detailed review of available literature, Andrén [9] found evidence for patch-area effects only in studies carried out in landscapes with ≤ 30% of remaining native vegetation. In the first targeted empirical test of this theoretical model, Pardini *et al.* [20] found evidence for patch-area effects on the richness and total abundance of the assemblage of habitat specialist small mammals only in a landscape characterized by intermediate levels of native vegetation cover (30%). Where vegetation clearance was high (10% native vegetation cover), an abrupt drop in landscape-wide richness and no evidence for patch-area effects on this assemblage was observed. However, despite this empirical support, direct evidence of reduced immigration into patches in landscapes with decreasing amounts of native vegetation – the principal mechanism driving patch-effects in the Andrén-Pardini model – is lacking [20].

In this study, we combine pattern and process orientated research approaches to investigate the demographic processes behind patterns of species abundance and persistence in patches in different landscape contexts [8], [21], [22]. We investigated patterns of population density and estimated demographic parameters of the endemic Gray Slender Mouse Opossum *Marmosops incanus* (Lund 1840, Didelphidae) in patches located in three 10000-ha Brazilian Atlantic forest landscapes which are characterized by different amounts of remaining forest (50, 30, and 10%). *M. incanus* is a small marsupial species (adult weight: 22 to 60 g in the study area; Barros, *unpublished data*) with a seasonal reproduction pattern (reproduction in the rainy season [23], [24]), peaks in abundances between December and June, and without strong inter-annual variation in abundance in the study area (Barros, *unpublished data*). It is a forest specialist species showing high habitat [25] as well as micro-habitat specificity [26], while also being relatively common and therefore amenable to population parameter estimation. Using two different data sets, we tested the hypotheses that: (1) patch-area effects on population density are apparent only at an intermediate level of forest cover, below which the species become extinct at the landscape scale (i.e. changes in population density of the model species are consistent with patterns of diversity observed for the assemblage of habitat specialist small mammals in the same landscapes [20]), and (2) immigration rates into patches are defined by landscape context, being higher in patches of the most forested landscape.

## Materials and Methods

### Study area

**Figure 1:**
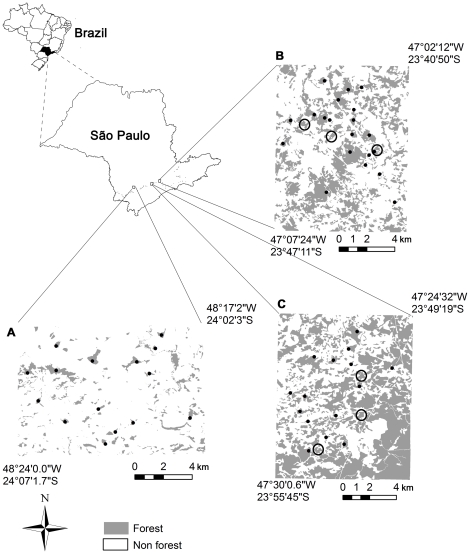
Distribution of forest patches in three fragmented landscapes in the Atlantic Plateau of São Paulo. (A) Fragmented landscape in Ribeirão Grande - Capão Bonito (10% forest cover), (B) fragmented landscape in Ibiúna (30% forest cover) and (C) fragmented landscape in Tapiraí - Piedade (50% forest cover). Forest patches are shown in gray. Dots: patches where *M. incanus* density was quantified (first dataset); circles: small patches where capture-recapture data was collected to estimate demographic parameters (second dataset).

Small mammals were sampled in three 10000-ha fragmented landscapes located in the Atlantic Plateau of São Paulo, Brazil, in the municipalities of Tapiraí - Piedade, Ibiúna, and Ribeirão Grande - Capão Bonito (Fig. 1). The entire region was originally covered with Atlantic Forest classified as “Lower Montane Atlantic Rain Forest” [27], which is currently reduced to patches of different sizes. In all three landscapes, patches consist of secondary forest and are surrounded mainly by pasture (48%, 44% and 50% of non-forest areas for Tapiraí - Piedade, Ibiúna, and Ribeirão Grande - Capão Bonito, respectively) and agriculture (26%, 20% and 35% of non-forest areas for Tapiraí - Piedade, Ibiúna e Ribeirão Grande - Capão Bonito, respectively). Altitudes are between 800 and 1000 m above sea level [28]. Annual rainfall is between 1222 and 1810 mm and mean minimum and maximum temperatures are 17.3°C and 28.4°C for the warm-wet season (October to March) and 12.1°C and 24.9°C for the cool-dry season (April to September). The three landscapes are similar in terms of topography, relief, climate, and type of human occupation, but vary in the proportion of remaining forest cover from 49% in Tapiraí – Piedade (hereafter referred to as 50%), to 31% in Ibiúna (hereafter 30%) and 11% in Ribeirão Grande - Capão Bonito (hereafter 10%, Fig. 1) and thus in patch and gap characteristics. The landscape with 50% forest cover has the highest percentage of the landscape covered by the largest patch, highest mean patch size, and lowest mean distance to the nearest patch, whereas the landscape with 10% forest cover presents the lowest values for these variables [20].

### Sampling design and Data Collection

#### Ethics Statement

Trapping and handling were approved by IBAMA – Instituto Brasileiro do Meio Ambiente e dos Recursos Naturais Renováveis (permissions 57/02 - IBAMA/SP, 11/04 - IBAMA/SP, 168/2004 - CGFAU/LIC, 237/2005 - CGFAU/LIC, 262/2006 – COFAN, 11577-2 - IBAMA/SP, and 11577-4 - IBAMA/SP) and conformed to guidelines sanctioned by the American Society of Mammalogists Animal Care and Use Committee. Because our study involved only the capture, handling for marking and the immediate release of a small marsupial in the field, it did not receive an approval from the Ethics Committee of the Institute of Biosciences - University of São Paulo (Comissão de Ética em Uso de Animais Vertebrados em Experimentação– CEA - http://ib.usp.br/etica_animais.htm), which only requires approval for studies on vertebrates that include experimentation (e.g. maintenance in captivity, injection of drugs, or surgery).

#### Patch-area effect on population density

We sampled 50 forest patches, 15 of which (3 to 145 ha) were located in the landscape with 50% forest cover, 20 in the landscape with 30% (2 to 374 ha) and 15 in the landscape with 10% (6 to 106 ha, Fig. 1). Surveyed forest patches were selected in order to ensure: similar vegetation structure and age (all patches consisted of secondary vegetation in intermediate stages of regeneration and were not subjected to disturbances such as fire, selective logging or cattle), extensive overlap in patch size among landscapes, guarantee a minimum distance among patches, avoid spatial segregation among similar-sized patches, and guarantee a minimum distance of the sampling site from the forest edge (30 m in small fragments but usually more than 50 m). Distances of sites to patch-edge, average distance to the nearest surveyed patch, as well as size and shape of surveyed patches did not differ significantly among landscapes [20]. The percentage of forest cover in an 800 m circumference around sampling sites within surveyed forest patches varied among landscapes (from 22 to 64% in the landscape with 50% of forest cover, 11 to 77% in the landscape with 30%, and 5 to 41% in the landscape with 10%), but was highly correlated with patch size in all three landscapes, with larger patches being surrounded by higher amounts of forest [20].

A standardized sampling protocol was used in all 50 sites. The protocol consisted of one 100-m long line of 11 60-L pitfall traps per site, with pitfall traps located 10 m from each other and connected by a 50-cm high plastic drift-fence. Four 8-day capture sessions were carried out at each site, two in each of two consecutive summers. In the Ibiúna landscape, capture sessions were conducted during the summers of 2001–2002 and 2002–2003, and in the other two landscapes during the summers of 2005–2006 and 2006–2007. There is no evidence that climatic conditions in the study region varied during the time of the study [29]. Sampling effort was concentrated in the wet season, since daily capture success with pitfall traps is higher at this time of the year [30]. Animals were marked with numbered tags on their first capture (Fish and small animal tag-size 1 – National Band and Tag Co., Newport, Kentucky). The number of individuals of *M. incanus* captured in the standardized area sampled in each of the 50 surveyed patches was used as an index of population density (hereafter density) and represents the first dataset.

#### Effect of landscape forest cover on immigration rates

Population demography was investigated in three small forest patches in each of the two most forested landscapes (50 and 30% forest cover), since the focus species was absent from the most deforested landscape (see results from the first data set). Patches were chosen to guarantee consistent differences in the amount of surrounding forest between landscapes, but to be otherwise similar (Fig. 1; Fig. S1):

1. similar size (30% landscape: 19.7 ha, 13.9 ha, 16.9 ha; 50% landscape: 18.3 ha, 14.8 ha, 17.1 ha);
2. similar distance among surveyed patches in each landscape (Fig. 1; Fig. S1);
3. similar number of connections to other patches by corridors (Fig. 1; Fig. S1);
4. similar forest structure (all patches are comprised of secondary forest in intermediate stage of regeneration);
5. different percentage of surrounding forest cover in order to maintain the overall percentage in the respective landscape (calculated in three radii around the center of each patch; 30% landscape 1 km: 28.7%±0.03; 1.5 km: 30.4%±0.03; 2 km: 30.6%±0.03; 50% landscape 1 km: 52.4%±0.07, 1.5 km: 51.0%±0.01, 2 km: 48.1%±0.03; all values mean among patches ± SD; Fig. S1).

A trapping grid of 2 ha was placed in each of the six patches (Fig. S2). Given the irregular shape of patches, one side of the grid was near to a forest edge in each of the six surveyed patches. Each grid consisted of 11 parallel 100-m long lines, 20 m from each other, with 11 trapping stations spaced every 10 m. In each trapping station one Sherman trap (size: 37.5 x 10.0 x 12.0 cm or 23.0 x 7.5 x 8.5 cm) was placed on the ground. In addition, five lines were also equipped with one 60-L pitfall trap per station, connected to each other by a 50-cm high plastic fence (similar to the trapping lines used for the investigation of patch-area effect on density). Two different trap types were used to maximize both capture and recapture rates, since pitfall traps result in higher capture rate and higher proportion of young individuals, while recapture rates are higher in Sherman traps [30]. All traps were baited with a mixture of sardines, peanut butter, banana, and manioc flour. Precautions were taken to minimize mortality in pitfall traps (bucket lids were used as a roof protecting from rain, small holes in the bottom facilitated drainage, and a styrofoam disc provided a dry surface in the event of accumulation of water).

Trapping design followed Pollock's robust design [31], [32], with short primary capture sessions assuming population closure, separated by relatively longer time periods, thereby allowing for open population processes. Animals were captured during five 5-day primary capture sessions with a 20-day interval between them, resulting in 26400 trap nights in total. This protocol was carefully established from our field experience with Atlantic forest small mammals, for which between-primary session recaptures tend to be very low due to short life-cycles. It represents a trade-off between guaranteeing open population processes between primary sessions on the one hand, and maximizing between-primary session recapture probabilities, which are a crucial requirement for the precise estimation of population parameters [33], on the other. Trapping within each primary session was carried out simultaneously in the three grids of each landscape, and consecutively (with no interval) between landscapes, to minimize the influence of weather and season on results and guarantee comparable estimates between landscapes. All five primary capture sessions were carried out between February and June of 2008. Captured animals were weighed, sexed, and marked with a numbered ear tag (small animal tags OLT – A. Hartenstein GmbH, Würzburg/Versbach, Germany) and released in the respective trapping location. The capture histories of *M. incanus* represent the second dataset used in the analyses.

### Data analyses

#### Patch-area effect on population density

**Figure 2:**
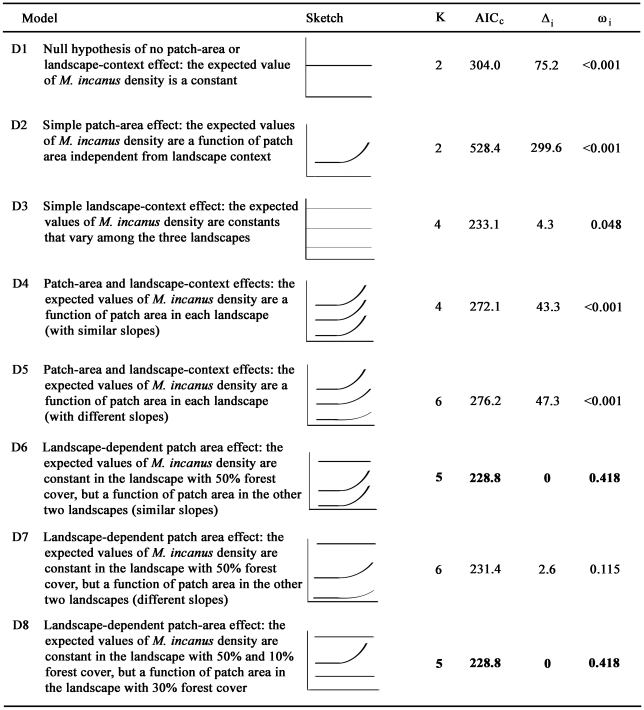
Set of candidate models to describe the variation in *M. incanus* density. Sketches represent the graphical description of model expectations on density of *M. incanus*. Y-axis: density, either representing a prediction independent of landscape context (single line, Models D1 and D2) or dependent on landscape context (three lines, Models D3 to D8); X-axis: patch area; K is the number of parameters in the model; AIC_c_ is the modified Akaike information criterion for small sample sizes; Δ_i_ is the difference between the AIC_c_ value of model D_i_ and the AIC_c_ value of the most parsimonious model; ω_i_ is the Akaike weight of model D_i_. Selected models (Models D6 and D8) with lowest AIC_c_ are shown in bold.

The data on the density of *M. incanus* in the 50 patches of the three fragmented landscapes was confronted with eight alternative models (Fig. 2), which were compared using an information-theoretic model selection approach [34]. Each candidate model is a combination of linear and/or constant functions, and corresponds to either (1) the lack of relationship between density and patch area or landscape context, (2) a positive relationship with patch area independent of landscape context, (3) a relationship with landscape context independent of patch area, or (4) a positive relationship with patch area in one, two, or three landscapes depending on landscape context (Fig. 2).

The log-likelihood of each model was calculated as the sum of the log-likelihoods of their component functions, with the maximum likelihood estimates for coefficients being the set of values that minimized the whole model negative log-likelihood (i.e. the sum of the negative log-likelihood of the component functions). As usual for count data, all models were fitted using log as the link function. Density was modeled as a Poisson variable in models with patch-area effect, and as a negative binomial variable in constant functions (i.e. without patch-area effects) due to high variance in these cases. Patch areas were converted by their logarithms (base 10). The Akaike Information Criterion corrected for small samples (AIC_c_) was calculated for each model and the plausibility of alternative models was estimated by the differences in their AIC_c_ values in relation to the AIC_c_ of the most plausible model (Δ_i_), where a value of Δ_i_ ≤ 2 indicates equally plausible models. All analyses were conducted in the R environment, version 2.13.0 [35].

#### Effect of landscape forest cover on immigration rates

To estimate the number of immigrants in each forest patch in each primary capture session, we first used capture-recapture histories of *M. incanus* to estimate abundance, apparent survival rates (including survival and emigration rates; hereafter survival rate) and population rates of change in program MARK version 6.0 [36]. Testing for population closure within primary capture sessions was performed with the program CloseTest (Stanley and Richards, Fort Collins Science Center, http://www.mesc.usgs.gov/Products/Software/clostest/) using Stanley and Burnham test for closure [37] when possible (in 24 of 30 primary capture sessions). Because only in five out of 24 primary capture sessions tests indicated violation of closure (χ^2^>5.15; *p*<0.05), and the number of trapping days per primary capture session was small (5 days), we assumed population closure within primary capture sessions.

Modeling of survival rates and population rates of change was based on Pollock's robust design models [31], [32] and Pradel Lambda models [38]. To assess whether survival rates (ϕ) and population rates of change (λ) of populations of *M. incanus* differ between landscape contexts or forest patches, we considered a set of nine candidate models, among which survival rate and population rate of change were either (1) constant between all patches independent of landscape context, (2) dependent on landscape context, or (3) different between patches independent of landscape context (Table 1):

**Table 1:** Models describing survival rates, population rates of change, capture rates and recapture rates of populations of *M. incanus* in two Atlantic forest landscapes with differing proportions of remaining forest (50% and 30%) in the Atlantic Plateau of São Paulo.

Model					K	AIC_c_	Δ_i_	ω_i_	Dev
I2	ϕ_(ls)_	λ_(ls)_	p_(pa+t)_	c_(pa+t)_	24	1211.5	0.0	0.377	1157.8
I1	ϕ_(.)_	λ_(.)_	p_(pa+t)_	c_(pa+t)_	22	1212.4	0.9	0.234	1163.7
I3	ϕ_(ls)_	λ_(.)_	p_(pa+t)_	c_(pa+t)_	23	1212.5	1.0	0.225	1161.4
I4	ϕ_(.)_	λ_(ls)_	p_(pa+t)_	c_(pa+t)_	23	1213.6	2.1	0.126	1162.5
I5	ϕ_(pa)_	λ_(ls)_	p_(pa+t)_	c_(pa+t)_	28	1218.1	6.6	0.013	1154.4
I6	ϕ_(ls)_	λ_(pa)_	p_(pa+t)_	c_(pa+t)_	28	1218.7	7.2	0.010	1155.0
I7	ϕ_(pa)_	λ_(.)_	p_(pa+t)_	c_(pa+t)_	27	1218.8	7.3	0.010	1157.6
I8	ϕ_(.)_	λ_(pa)_	p_(pa+t)_	c_(pa+t)_	27	1220.9	9.4	0.003	1159.7
I9	ϕ_(pa)_	λ_(pa)_	p_(pa+t)_	c_(pa+t)_	32	1224.1	12.6	<0.001	1149.8

In all models, capture rate (p) and recapture rate (c) are specific to the patch (pa) and the primary capture session (t). Survival (ϕ) and population rate of change (λ) are either considered constant (.), landscape-specific (ls), or patch-specific (pa). K is the number of parameters in the model; AIC_c_ is the modified Akaike information criterion for small sample sizes; Δ_i_ is the difference between the AIC_c_ value of Model I_i_ and the AIC_c_ value of the most parsimonious model; ω_i_ is the Akaike weight of Model I_i_; Dev is the deviance. Models are ranked by their AIC_c_-values.

Model I1 – Survival rate and population rate of change are constant between all patches independent of landscape context: this model represents a null hypothesis of no effects on *M. incanus* demographic parameters;

Model I2 – Survival rate and population rate of change depend on landscape context: the expected values of survival rate and population rate of change are similar within landscapes, but different between them;

Models I3 and I4 – Survival rate depends on landscape context and population rate of change is constant between all patches independent of landscape context (or vice versa): these models assume that the expected values of survival rate are similar within a landscape but different between them, while population rate of change is constant between patches independent of landscape context (Model I3) or vice versa (Model I4);

Models I5 and I6 – Survival rate differs between patches, and population rate of change depends on landscape context (or vice versa): these models assume that the expected values of survival rate differ between patches independent of landscape context, while population rates of change are similar within a landscape but different between them (Model I5) or vice versa (Model I6);

Models I7 and I8 – Survival rate differs between patches independent of landscape context and population rate of change is constant between patches (or vice-versa): these models assume that the expected values of survival rate and population rate of change are both independent of landscape context, but the values of survival rate depend on patches and those of population rate of change are constant (Model I7) or vice versa (Model I8);

Model I9 – Survival rate and population rate of change differ between patches independent of landscape context: this model assumes that the expected values of survival rate and population rate of change are both independent of landscape context, but differ between patches.

Based on experience during field work, capture probability (p) and recapture probability (c) were considered different between patches and dependent on primary capture sessions, while constant within primary capture sessions in all candidate models. Because our dataset did not allow for heavily parameterized models, the estimation of population size (N) was conditioned out of the likelihood using Huggins'-closed-capture models within primary capture sessions [39]. We used the logit-link function for estimation of survival, capture, and recapture rates. A log-link function was used for the estimation of population rate of change. Model selection procedure with the Akaike Information Criterion corrected for small samples (AIC_c_) was the same as described above for the models describing population density. In order to account for model selection uncertainty, we used the AIC_c_ weights (ω_i_) to calculate weighted averages [34], [40] of population sizes and survival rates, which were used in the estimation of number of immigrants per capture session.

For the estimation of the number of immigrants per capture session, the estimated number of surviving individuals from the preceding capture session was subtracted from the estimated population size of adults (i.e. estimated population size without young individuals considered to be born in the trapping area and therefore not immigrated) in sessions two to five [41]:
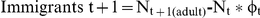



With N_t+1(adult)_: estimated population size of adults at time t+1

N_t_: estimated total population size at time t

φ _t_ : estimated survival rate between t and t+1

Model averaged estimates of total population size (N_t_) and survival (φ_t_) were obtained by using the complete capture histories of *M. incanus* for parameter estimation in program MARK. Model averaged estimates of population size of adults (N_t+1(adult)_) were obtained by using reduced capture histories of *M. incanus* excluding young individuals which were considered born in the trapping area and therefore not immigrated. Identification of young individuals was based on tooth eruption patterns [42]. Individuals with up to the second molar erupted in the upper jaw were classified as young individuals, since individuals with the third molar erupted are considered sexually active and therefore potentially moving longer distances [42]. The numbers of immigrants per primary capture session were compared between landscapes using mixed-effect models to control for dependence among primary trapping sessions in the same forest patch (fixed factor: landscape; random factors: forest patch and primary trapping session). The significance of difference between landscapes was verified by Markov chain Monte Carlo simulation (n = 10,000). Statistical analyses were conducted in the R environment, version 2.13.0 [35].

## Results

### Patch-area effect on POPULATION density

We captured 189 individuals of *M. incanus* distributed across the 15 patches of the 50% landscape, and 178 individuals distributed in 19 out of 20 patches of the 30% landscape, while no individuals were captured in the surveyed patches of the 10% landscape. It is important to note that *M. incanus* was captured in continuously-forested areas adjacent to each of the three fragmented landscapes [20], indicating that its absence in the 10% landscape is not due to biogeographical differences among landscapes. Mean density among surveyed patches was 12.9±9.6 in the 50% landscape and 8.9±4.8 in the 30% landscape (mean ± SD, respectively).

**Figure 3:**
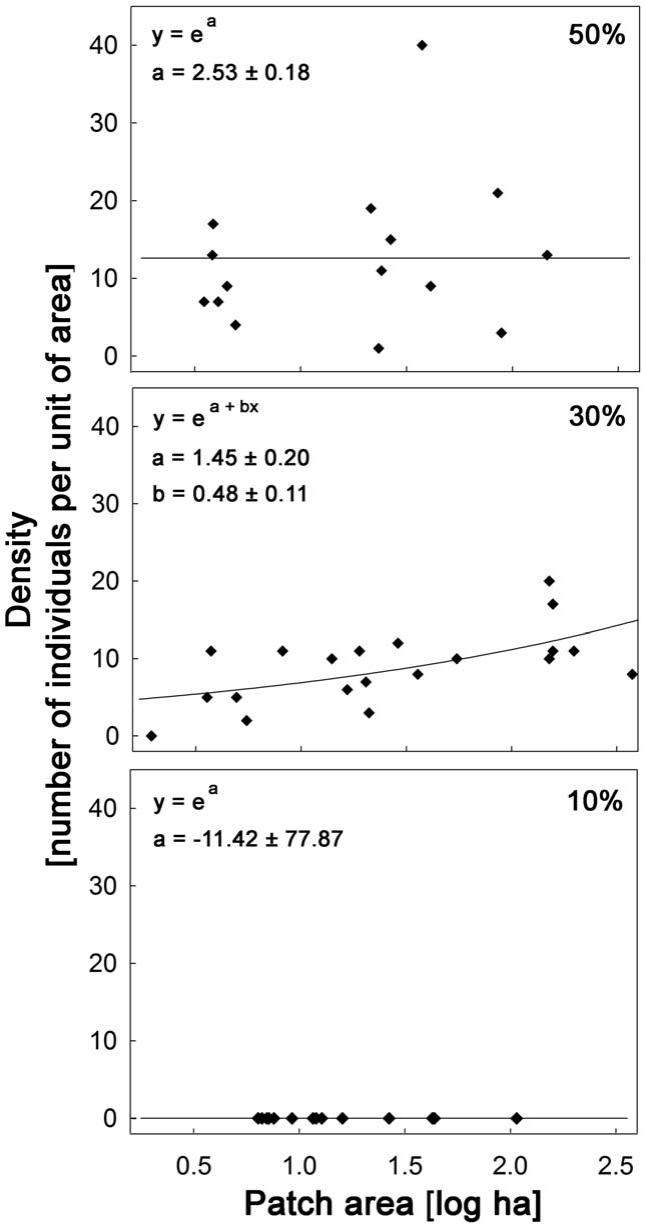
Observed (diamonds) and estimated (lines, Model D8) densities of *M. incanus*. Percentages (50%, 30%, 10%) represent the proportion of forest cover in three Atlantic forest landscapes in the Atlantic Plateau of São Paulo. Model equations and parameter estimates *+/-* SE are given in the upper left corners.

Two models were selected as equally plausible to explain the variation in *M. incanus* density (Model D6 and Model D8, Fig. 2). Both predicted a landscape-dependent patch-area effect only in the landscape with intermediate forest cover (Model D8, Fig. 3) or in the two most deforested landscapes (Model D6). However, in this case Model D6 is redundant with Model D8, since the estimated positive slope coefficient of the patch-area effect on density in the 10% landscape is a value close to zero and the predicted function is indistinguishable from a constant function.

### Effect of landscape forest cover on immigration rates

We captured 47 individuals of *M. incanus* 142 times in the three patches of the most forested landscape (50% forest cover), and 31 individuals 103 times in the three patches of the landscape with intermediate forest cover (30%). The best model among the demographic candidate models predicted both survival rates and population rate of change to be different between landscapes, but similar within each landscape, i.e. dependent on landscape context (Model I2, Table 1). Two models with fewer parameters were selected as equally plausible to the best model. The second selected model predicted constant survival rates and population rates of change between patches independent of landscape context (Model I1, Table 1), and the third model predicted survival rates to be dependent on landscape context, and population rates of change to be constant between patches independent of landscape context (Model I3, Table 1).

**Figure 4:**
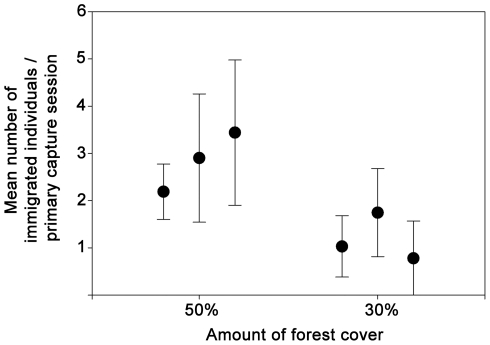
Mean number of immigrated individuals of *M. incanus* per capture session (+/- SE). Immigration into small patches was estimated at two Atlantic forest landscapes with different proportions of remaining forest (50% and 30%) in the Atlantic Plateau of São Paulo.

Weighted average estimates of population sizes are congruent with results from the first data set, resulting in higher values in patches of the most forested landscape (Table 2, Table S1). In this landscape, weighted averaged estimates of apparent survival as well as estimates of population rate of change were also slightly higher (Table 3). Further, numbers of immigrated individuals per capture session were significantly higher in patches of the most forested landscape (Fig. 4, *p* = 0.043).

**Table 2:** Model-averaged estimates of population sizes (N) of *M. incanus* in small patches of the landscape with 50% of remaining forest cover (patches 1 to 3) and of the landscape with 30% of remaining forest cover (patches 4 to 6), in primary capture sessions 1 to 5 between February and June 2008.

Amount of forest	Patch number	Primary capture session	N	Lower 95% CI	Upper 95% CI
50%	1	1	3.29	3.02	7.60
	1	2	4.47	2.41	16.73
	1	3	5.09	3.35	15.42
	1	4	5.13	5.01	7.69
	1	5	6.31	6.02	10.10
	2	1	3.08	3.00	5.21
	2	2	8.04	5.64	19.51
	2	3	5.25	4.19	12.33
	2	4	7.04	7.00	8.38
	2	5	8.10	8.00	10.31
	3	1	8.44	8.03	14.08
	3	2	13.23	8.70	29.79
	3	3	8.90	6.63	19.33
	3	4	8.10	8.00	10.26
	3	5	7.19	7.01	10.22
30%	4	1	2.01	2.00	2.55
	4	2	4.80	4.09	10.94
	4	3	3.26	3.02	6.71
	4	4	2.00	2.00	2.16
	4	5	1.00	1.00	1.20
	5	1	4.04	4.00	5.57
	5	2	1.37	1.03	5.71
	5	3	4.71	4.08	10.46
	5	4	4.01	4.00	4.55
	5	5	4.02	4.00	4.96
	6	1	5.00	5.00	5.05
	6	2	7.08	7.00	9.02
	6	3	6.02	6.00	6.89
	6	4	2.00	2.00	2.00
	6	5	1.00	1.00	1.01

**Table 3:** Model-averaged estimates of apparent survival rates and population rates of change of populations of *M. incanus* in small patches of the landscape with 50% of remaining forest cover (patches 1 to 3) and of the landscape with 30% of remaining forest cover (patches 4 to 6) between February and June 2008.

Parameter	Patch number	Estimate	Lower 95% CI	Upper 95% CI
Apparent survival rate 50% landscape	1	0.61	0.48	0.74
	2	0.62	0.48	0.74
	3	0.62	0.48	0.73
Apparent survival rate 30% landscape	4	0.50	0.35	0.66
	5	0.51	0.35	0.66
	6	0.50	0.34	0.66
Population rate of change 50% landscape	1	1.04	0.87	1.22
	2	1.04	0.87	1.21
	3	1.04	0.87	1.20
Population rate of change 30% landscape	4	0.93	0.37	1.00
	5	0.93	0.37	1.00
	6	0.93	0.38	1.00

## Discussion

Landscape-level study designs (i.e. not only considering several patches in each landscape but also replicated landscapes) are challenging given the logistical difficulties of obtaining robust field data in a large number of sites. Our patch-level study, although limited by the small number of surveyed landscapes, provides evidence that population dynamics in patches depend on landscape context. Lower population sizes and lower number of immigrants of *M. incanus* into small patches in the landscape with 30% forest cover compared to patches in the most forested landscape are congruent with predictions from simulation models on the effects of landscape-wide habitat cover [10], [14], [15]. In combination with patch-area effects on *M. incanus* density being observed only in the landscape with 30% forest cover and the absence of the species in the most deforested landscape, these results are in accordance with the idea that variations in immigration rates represent the mechanism behind patch-area effects [9], [20] and species extinction thresholds [13], [18], [19], [43] across landscapes with differing proportions of remaining native vegetation. Thus, the results on the demography of a forest specialist species reported here are congruent with the patterns of diversity observed for the whole assemblage of forest specialist small mammals across the same landscapes [20].

Although previous studies as far as we are aware did not investigate the effects of landscape context on immigration rates, immigration has consistently been shown to increase population size in patches both in field studies and simulation models [44]–[47]. Differences in population size among patches could also be caused by varying *in situ* recruitment rates, which we were not able to estimate due to low capture probabilities of young individuals. However, the observed number of young individuals in patches was similar among landscapes, suggesting that differences in population size were primarily driven by variation in immigration rates. In landscapes with a high proportion of remaining habitat and small distances among patches, high dispersal increases population size in patches, thereby rescuing small populations in small patches from the risk of local extinction [16], [20]. As a consequence, population densities should be similar among patches irrespective of patch size, as we observed in this study. Given high dispersal between patches in the most forested landscape, the population of *M. incanus* can be classified as a patchy population (i.e. high dispersal rates between sub-populations effectively forming one population [48]). Indeed the confidence interval of the estimated population rate of change in small patches in this landscape encompasses the value of one, indicating that the higher immigration rate supports a constantly high population size.

On the other hand, not only was there a clear patch-area effect on the density of *M. incanus* in the landscape with intermediate forest cover (30%), but also the number of immigrants decreased significantly in small patches of this landscape in comparison to patches of the more forested landscape, despite the relatively small sample size and thus statistical power. Supposedly, the decrease in immigrants led to the comparatively lower population sizes in these patches. Further, the confidence intervals of the population rate of change only marginally encompass the value of one, indicating a slight decline in population size during the study. Although the time frame of the study is too short to infer general population trends, low population rate of change is considered a typical indicator of extinction risk [17], [49], and this short-term population decline in these already small populations may indeed indicate an increased extinction risk from stochastic events. The lower immigration rates into small patches of this landscape with intermediate forest cover is presumed to be caused by increased inter-patch distances, which should increase the time spent in the matrix during dispersal events and thereby increase mortality and reduce dispersal success [50]–[53]. In the Atlantic forest, predation of forest marsupials by snakes and birds of prey has been shown to be higher in the open matrix than in forest patches [54], [55]. The time an individual spends in the matrix might be additionally increased in landscapes with decreased native vegetation cover through reduced perception probability of neighboring suitable habitat patches [56], [57]. Reduced or hindered neighborhood perception might result in non-optimal movements and hence longer times in the matrix and higher predation risk [58]. With a lower number of immigrants, but no complete isolation of local populations in patches, within-patch processes (births and deaths) should become relatively more important than between-patch processes, and the population can be described as a meta-population [59], [60].

Increased isolation due to the reduction in the number of immigrants might also affect genetic diversity [61], [62]. In an investigation of genetic diversity in functional markers (Major Histocompatibility Complex, MHC) in the 30% forest cover landscape, Meyer-Lucht *et al.* [63], [64] found surprisingly low numbers of alleles and associated higher parasite loads in populations of *M. incanus* compared to a sympatric didelphid marsupial, *Gracilinanus microtarsus*, which also occupies savanna-like habitats [65], [66], and is considered less dependent on forest. Furthermore, a study on genetic variation based on non-functional markers (microsatellites) of populations of *M. incanus* in the same two landscapes found lower genetic diversity in populations of the less forested landscape (30%) as expected when dispersal between populations is reduced (Balkenhol, N., *unpublished data*).

Our results are also congruent with a fragmentation threshold sensu Andrén [9], [12], [20], namely a threshold in habitat amount below which patch-area effects become apparent, with a similar value to that proposed in a review of independent studies on birds and mammals (≤30% [9]). Furthermore, our results are in accordance with the notion that this threshold might be a first step leading to a drastic decrease in the probability of species persistence at the landscape scale (extinction threshold [18], [19], [43], [67]), as recently proposed by Pardini *et al.* [20]. Given the similarity between *M. incanus* and several forest specialist small mammals in ecological requirements [25], [26] and body size, the reduction in immigration rates, the variation in the strength of patch-area effects on population density, and the increased extinction probability across landscapes with decreasing proportion of forest cover are likely valid for other Atlantic forest species. The simultaneous extinction of forest specialist species in highly deforested landscapes would lead to an abrupt drop in gamma diversity [20], [68].

Our study points out the value of single-species, process-oriented studies in understanding multi-species, larger-scale patterns [22]. While investigations of the effects of habitat fragmentation on population demography have focused almost exclusively on patches in single landscapes, our study provides empirical evidence for the dependence of population dynamics in patches on landscape context. By revealing immigration rates as a plausible candidate for the underlying mechanism causing both patch-area effects and species extinction thresholds, this study is in accordance with theoretical models proposing landscape-wide habitat cover as the main determinant of species persistence in fragmented landscapes [13], [18], [19]. This highlights the relevance of policies that promote landscape-wide preservation of native vegetation [68], such as the mandatory legislation to maintain native vegetation at a fixed percentage of private properties in Brazil (Forest Act [69], [70]). Such policies are especially important in highly endangered biomes like the Atlantic forest, where more than 80% of all forest patches, representing more than 20% of the remaining forest area, are smaller than 50 ha [71].

Future research should investigate the influence of landscape-wide habitat amount on population demography in species varying in ecological traits, such as the degree of habitat specialization and dispersal ability, and in other ecosystems, as well as focus on the consequences of reduced immigration on genetic diversity, fitness and mating, and social systems.

## Supporting Information

Figure S1 **Detail of distribution of forest patches investigated for estimation of demographic parameters of** ***M. incanus.*** (A) Fragmented landscape in Tapiraí - Piedade (50% forest cover) and (B) fragmented landscape in Ibiúna (30% forest cover). Forest patches are shown in gray. Dots: small forest patches where capture-recapture data was collected to estimate demographic parameters of populations of *M. incanus* (second dataset); circles: buffers around forest patches of a = 1 km, b = 1.5 km, and c = 2 km in which percentage of forest cover matches forest cover of the entire landscape.
(TIF)

Figure S2 **Scheme of the 2-ha trapping grids used to capture** ***M. incanus*** **.** Identical grids were installed at six forest patches located in two Atlantic forest landscapes with different proportions of remaining forest (50% and 30%) in the Atlantic Plateau of São Paulo (second dataset). White rectangles: Sherman traps; black circles: pitfall traps; black lines: plastic fence connecting pitfall traps of one line.
(TIF)

Table S1 **Model-averaged estimates of population sizes of adults (N_(adult)_) of** ***M. incanus*** **in small patches of the landscape with 50% of remaining forest cover (patches 1 to 3) and of the landscape with 30% of remaining forest cover (patches 4 to 6), in primary capture sessions 2 to 5 between February and June 2008.**
(DOC)
