# Supplementary figures and images for: Immigration Rates in Fragmented Landscapes – Empirical Evidence for the Importance of Habitat Amount for Species Persistence
Source: PLoS One. 2011 Nov 18;6(11):e27963. doi: 10.1371/journal.pone.0027963 (PMC3220714; doi:10.1371/journal.pone.0027963)

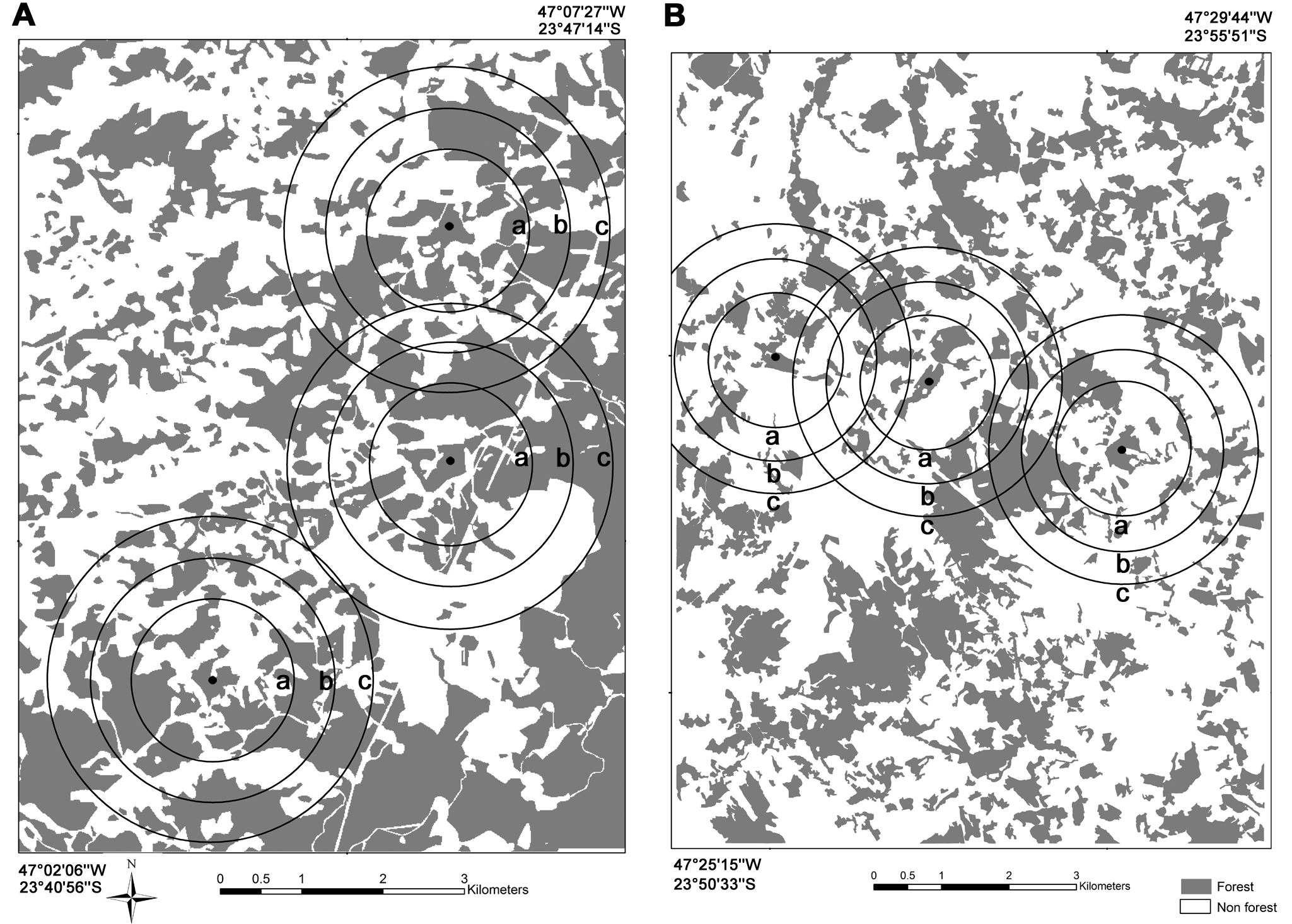

Supplement: Figure S1 — Detail of distribution of forest patches investigated for estimation of demographic parameters of M. incanus. (A) Fragmented landscape in Tapiraí - Piedade (50% forest cover) and (B) fragmented landscape in Ibiúna (30% forest cover). Forest patches are shown in gray. Dots: small forest patches where capture-recapture data was collected to estimate demographic parameters of populations of M. incanus (second dataset); circles: buffers around forest patches of a = 1 km, b = 1.5 km, and c = 2 km in which percentage of forest cover matches forest cover of the entire landscape. (TIF) [file pone.0027963.s001.tif]

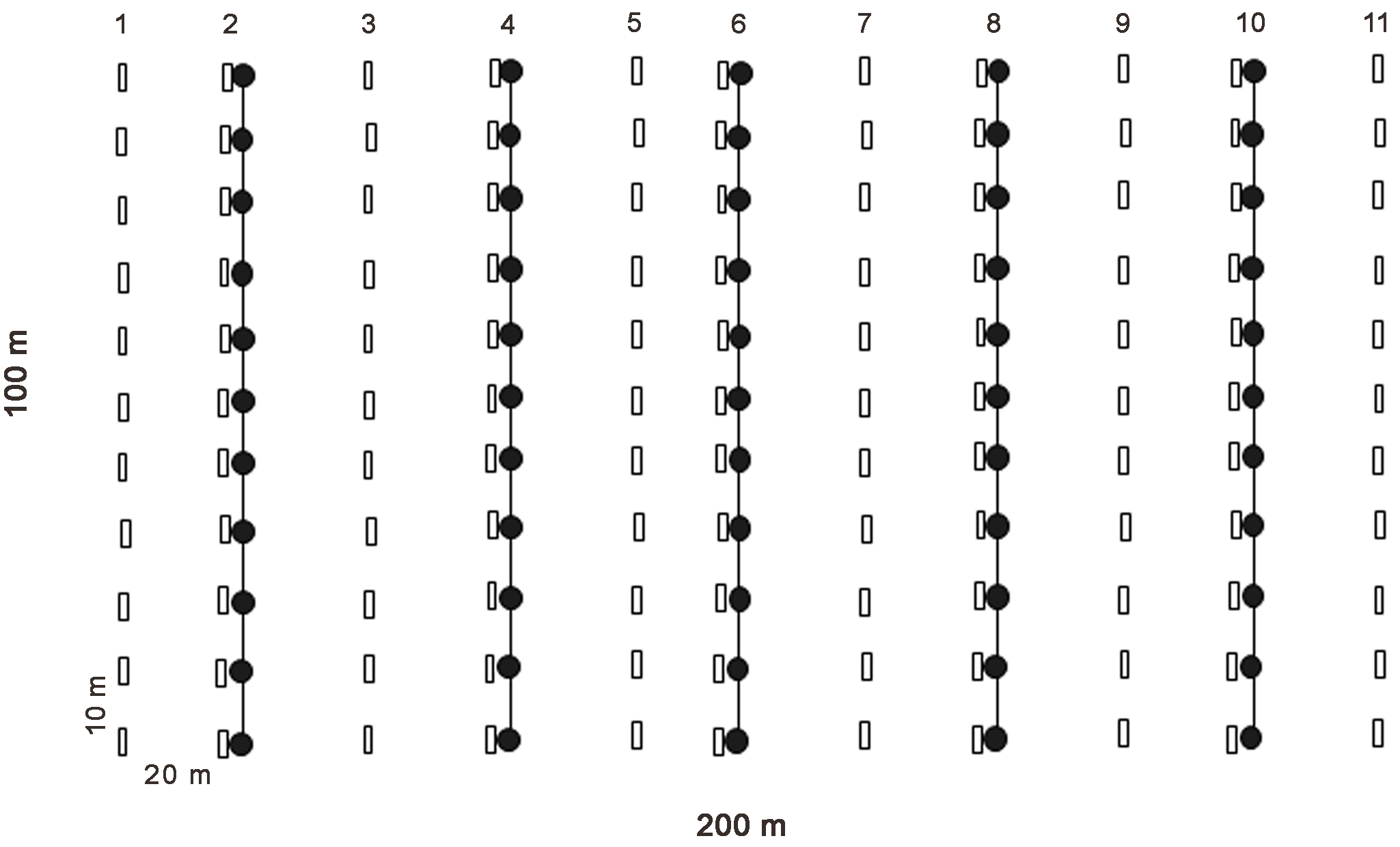

Supplement: Figure S2 — Scheme of the 2-ha trapping grids used to capture M. incanus . Identical grids were installed at six forest patches located in two Atlantic forest landscapes with different proportions of remaining forest (50% and 30%) in the Atlantic Plateau of São Paulo (second dataset). White rectangles: Sherman traps; black circles: pitfall traps; black lines: plastic fence connecting pitfall traps of one line. (TIF) [file pone.0027963.s002.tif]
