# Supplementary material for: Immigration Rates in Fragmented Landscapes – Empirical Evidence for the Importance of Habitat Amount for Species Persistence
Source: PLoS One. 2011 Nov 18;6(11):e27963. doi: 10.1371/journal.pone.0027963 (PMC3220714; doi:10.1371/journal.pone.0027963)
Supplement: Table S1 — Model-averaged estimates of population sizes of adults (N(adult)) of M. incanus in small patches of the landscape with 50% of remaining forest cover (patches 1 to 3) and of the landscape with 30% of remaining forest cover (patches 4 to 6), in primary capture sessions 2 to 5 between February and June 2008. (DOC) [file pone.0027963.s003.doc]

**Supporting information**

**Table S1.**

| Amount of forest | Patch number | Primary capture session | N_(adult)_ | Lower 95% CI | Upper 95% CI |
| --- | --- | --- | --- | --- | --- |
| 50% | 1 | 2 | 5.02 | 2.52 | 19.47 |
|  | 1 | 3 | 3.36 | 2.19 | 11.84 |
|  | 1 | 4 | 5.12 | 5.01 | 7.58 |
|  | 1 | 5 | 6.32 | 6.02 | 10.16 |
|  | 2 | 2 | 7.01 | 4.60 | 19.11 |
|  | 2 | 3 | 3.90 | 3.11 | 10.08 |
|  | 2 | 4 | 7.03 | 7.00 | 8.29 |
|  | 2 | 5 | 8.10 | 8.00 | 10.30 |
|  | 3 | 2 | 13.12 | 7.47 | 31.66 |
|  | 3 | 3 | 9.09 | 6.67 | 20.27 |
|  | 3 | 4 | 8.11 | 8.01 | 10.36 |
|  | 3 | 5 | 7.23 | 7.01 | 10.58 |
|  |  |  |  |  |  |
| 30% | 4 | 2 | 3.92 | 3.11 | 10.59 |
|  | 4 | 3 | 3.29 | 3.02 | 6.98 |
|  | 4 | 4 | 2.00 | 2.00 | 2.18 |
|  | 4 | 5 | 1.00 | 1.00 | 1.24 |
|  | 5 | 2 | 1.46 | 1.04 | 6.32 |
|  | 5 | 3 | 4.65 | 4.07 | 10.18 |
|  | 5 | 4 | 4.01 | 4.00 | 4.49 |
|  | 5 | 5 | 4.02 | 4.00 | 4.92 |
|  | 6 | 2 | 4.12 | 4.01 | 6.60 |
|  | 6 | 3 | 6.03 | 6.00 | 7.26 |
|  | 6 | 4 | 2.00 | 2.00 | 2.01 |
|  | 6 | 5 | 1.00 | 1.00 | 1.01 |
